# Symptoms of Diseased Elbow-Joint—Intense Pain—Operation—Abscess in the Olecranon, with Necrosed Bone—Recovery

**Published:** 1856-09

**Authors:** 


### RECORD OF MEDICAL SCIENCE.

*Symptoms of diseased Elbow-Joint—Intense Pain—Operation—Abscess in the Olecranon, with Necrosed Bone—Recovery.* Under the care of Mr. Curling.

Benjamin Smith, aged 33, by trade an engineer, of steady habits, and usually enjoying good health, was admitted into the London Hospital, on March 29, 1853. The particulars of his affection, both in its history and subsequent progress, were carefully recorded by Mr. John Langston, one of Mr. Curling's dressers, of whose notes the following is an abstract:—

March 29.—He applies on account of what looks like disease of the left elbow-joint. There is some swelling around the whole joint, but it is chiefly limited to the posterior part, where the integuments are tense and red. The arm is held rigidly flexed at a right angle, and there is exquisite tenderness about the part, more especially from the olecranon, down the course of the ulna, the slightest touch on which causes him to flinch. He states that for some weeks past he has had most severe and constant pain in this part, and his haggard appearance fully corroborates his account. He states that he was in usual health up to January 15, and had but recently returned from a voyage to the Cape of Good Hope. On the day mentioned, he was attacked by what seemed to be acute rheumatism which confined him to bed for more than a week, and during which several of the joints were much swollen. The general symptoms subsided under treatment, and with them the pain in all the joints, excepting the left elbow, in which it steadily increased. At first there was neither swelling nor redness about this joint, but only an intolerable aching pain. For a week or two before admission, he had attended as an out-patient, under the care of Mr. Ward, and iodine paint had been applied to the part. There were two circumstances in the account of his previous illness which were of importance to be borne in mind in connexion with his present affection, viz., that he had on two occasions prior to the one just mentioned suffered from acute rheumatism, and that at the age of 2, he had, in consequence of a severe blow, had an attack of severe inflammation about the same elbow as that now affected. This attack had been sufficiently urgent to necessitate his attendance for some time as an out-patient at a Hospital. Leeches and blisters were applied, and an abscess opened, but eventually he got quite well, and had perfect use of the joint. From the state of the parts and the character of the pain, Mr. Curling inferred that the disease was rather in the bone itself than the joints; as no movements or manipulation could, however, be borne, the diagnosis was only conjectural.

Ordered six leeches, followed by poultice. Low diet.

March 31.—The pain continues as severe as ever, but with some paroxysmal augmentations of severity. It almost entirely prevents sleep. He is to have half-a-grain of the acetate of morphia every night at bed-time.

April 3.—No relief. He has lost appetite, and is rapidly losing flesh and strength. It now appears that, prior to admission, he had of his own accord taken large doses of laudanum for the relief of the pain. Under the influence of chloroform, Mr. Curling this morning made a deep puncture over the olecranon, but no pus was obtained. It was found that the joint had perfect mobility while the patient was unconscious, and no signs of disease within the articulation could be discovered. Two leeches were to be applied every other night, and the dose of morphia increased to a grain.

11th.—The severity of the pain and its nocturnal exacerbations have so far enfeebled the patient that he is now scarcely able to stand without assistance. Nothing whatever has been gained by the treatment. He is to discontinue the leeches, and have a strong opiate lotion applied to the part.

16th.—A blister over the external condyle is to be applied. Morphia in grain doses every six hours.

20th.—It has been determined in consultation to make more free incisions over the ulna.

21st.—This morning Mr. Curling made a free incision along the course of the ulna, from above downwards, for a length of nearly two inches. No pus was obtained. The limb is confined to an angular splint, and the man is to have six ounces of wine a-day. Continue the morphia.

26th.—There has been a slight relief, in some respect, until to-day, when the pain is as severe as ever. The wounds are healing, and do not discharge more pus than their own surfaces supply.

27th.—A general consultation was held on the case to-day. Mr. Luke suggests, and Mr. Curling is inclined to agree with him, that there is probably matter pent up within the bone. Others are of opinion that the intense pain depends upon commencing ulceration of the articular cartilages, and advise a course of mercury. As the patient has been a little better during the last week it is decided to wait.

May 5.—The pain has been very intense ever since the last note. The man describes it as like plunging a number of needles into the part. A course of the iodide is to be tried. ℞ Pot. iodid. gr. iij; dec. sars. ℥i ter die.

12th.—The dose of the iodide is to be increased up to 5 grains.

13th.—Catarrhal symptoms from the iodide have set in with some severity. The dose is to be taken but twice a-day.

14th.—Catarrh more severe. The iodide to be suspended.

25th.—No permanent relief whatever having been obtained by any of the various plans of treatment which have been tried, it has at length been decided to trephine the head of the ulna. The condition of the part is much the same as at the time of admission, excepting that the swelling is more decidedly limited to the back of the joint, and the parts overlying the ulna. The incisions made have wholly healed, excepting one small sinus which leads down to the bone; but from this there is very little discharge indeed, and the surface of the bone is not bare to any extent.

*Operation.*—The man having been put under the influence of chloroform, an incision two inches long from near the extremity of the ulna downwards, and a second inwards at right angles with the first, were made. The skin having been dissected up, the bone was laid bare. On examining the part to which the sinus led, a very minute opening was discovered, leading into the bone. This was taken as a guide for the application of the trephine, and a small circle having been moved by the latter, the cavity of an abscess containing some necrosed fragments was opened. From this cavity two portions of dead and perfectly loose bone—one about the size of a nut, and the other that of a horse-bean—were removed. This seemed satisfactory; and, as the cavity very nearly opened into the joint above, no prolonged exploration was made. The elbow was wrapped in wet lint, and the arm confined in an angular splint.

26th.—The man has had excessive pain, and, in spite of increased doses of morphia, has had no sleep.

27th.—No rest has been obtained. The elbow is poulticed.

29th.—The severe pain continues. A drachm dose of laudanum is to be taken every four hours, instead of the morphia.

June 2.—The pain is greatly abated; the patient has had good sleep, and is much improved.

7th.—Rapidly improving in every particular, and now able to walk out in the garden. Wound healthy in appearance, and discharging freely.

15th.—The wound is now nearly healed, and the patient is free from pain. The arm is still, however, kept in the flexed position, though it is quite certain that the joint is not diseased. The man is is to leave the Hospital, and go down to the Ramsgate Sea-bathing Infirmary very shortly.

After remaining about five weeks at Ramsgate, during which the wound quite healed, the man was discharged. He had quite regained his health, and was free from pain; the motions of the elbow had, however, not been then perfectly recovered, though there seemed every reason to expect that they shortly would be so. He has not since been seen.—*Medical Times and Gazette.*

---

*Symptoms of Diseased Elbow-Joint—Removal of Necrosed Bone from the Olecranon—Partial Recovery.* Under the care of Mr. Curling.

